# Modelling individual infancy growth trajectories to predict excessive gain in BMI z-score: a comparison of growth measures in the ABCD and GECKO Drenthe cohorts

**DOI:** 10.1186/s12889-023-17354-4

**Published:** 2023-12-05

**Authors:** Anton Schreuder, Eva Corpeleijn, Tanja Vrijkotte

**Affiliations:** 1 Department of Public and Occupational Health, Amsterdam UMC, Amsterdam Public Health Research Institute, University of Amsterdam, Amsterdam, The Netherlands; 2 Leiden Institute of Advanced Computer Science, Leiden University, Leiden, The Netherlands; 3 Department of Epidemiology, University of Groningen, University Medical Center Groningen, Groningen, The Netherlands

**Keywords:** Risk, Prediction, Model, Body mass index, Overweight, Infant, Child, Growth, Body-weight trajectory, Mass screening

## Abstract

**Background:**

Excessive weight gain during childhood is a strong predictor for adult overweight, but it remains unknown which growth measures in infancy (0–2 years of age), besides predictors known at birth, are the strongest predictors for excessive weight gain between 2 and 5–7 years of age.

**Methods:**

The Amsterdam Born Children and their Development (ABCD) study formed the derivation cohort, and the Groningen Expert Center for Kids with Obesity (GECKO) Drenthe study formed the validation cohort. Change (Δ) in body mass index (BMI) z-score between 2 and 5–7 years was the outcome of interest. The growth measures considered were weight, weight-for-length (WfL), and body mass index (BMI). Formats considered for each growth measure were values at 1, 6, 12, and 24 months, at the BMI peak, the change between aforementioned ages, and prepeak velocity. 10 model structures combining different variable formats and including predictors at birth were derived for each growth measure, resulting in 30 linear regression models. A Parsimonious Model considering all growth measures and a Birth Model considering none were also derived.

**Results:**

The derivation cohort consisted of 3139 infants of which 373 (11.9%) had excessive gain in BMI z-score (> 0.67). The validation cohort contained 2201 infants of which 592 (26.9%) had excessive gain. Across the 3 growth measures, 5 model structures which included measures related to the BMI peak and prepeak velocity (derivation cohort area under the curve [AUC] range = 0.765–0.855) achieved more accurate estimates than 3 model structures which included growth measure change over time (0.706–0.795). All model structures which used BMI were superior to those using weight or WfL. The AUC across all models was on average 0.126 lower in the validation cohort. The Parsimonious Model’s AUCs in the derivation and validation cohorts were 0.856 and 0.766, respectively, compared to 0.690 and 0.491, respectively, for the Birth Model. The respective false positive rates were 28.2% and 20.1% for the Parsimonious Model and 70.0% and 74.6% for the Birth Model.

**Conclusion:**

Models’ performances varied significantly across model structures and growth measures. Developing the optimal model requires extensive testing of the many possibilities.

**Supplementary Information:**

The online version contains supplementary material available at 10.1186/s12889-023-17354-4.

## Introduction

The proportion of children with overweight has been increasing world-wide for over the past half century [1, 2]. Being overweight during adolescence often persists into adulthood and increases the lifetime risk for a wide range of diseases [3–7]. In turn, higher body mass index (BMI) percentile at the age of 6 years, rapid growth between the ages of 2 and 7 years, and an earlier adiposity rebound are strong predictors of overweight and obesity in adolescence and adulthood [8–17]. Barker et al. [18] described that the risk of coronary heart disease in adults was more strongly related to the increase of BMI in childhood than to the BMI attained at any particular age. Therefore, optimization of infant and child growth could lead to substantial reductions in adverse health outcomes in later life.

The classification of overweight and obesity are based on BMI z-scores above a certain threshold [19]. However, BMI is not a perfect indicator of body fatness so this outcome measures does not perfectly identify the target population [20]. Given the role of height-squared in calculating BMI, broader individuals are more likely to surpass the threshold despite having a lower fat or higher muscle percentage. This is why a strong predictor for high future BMI z-score is a high prior BMI z-score, though this usually indicates a stable growth trajectory in children. However, children who experienced catch-up growth to compensate for intrauterine growth retardation were also more vulnerable to future overweight and unfavourable body composition, i.e., relative greater increase in fat compared to lean mass [12, 14, 15, 21]. The same applies to children without growth retardation but with body weights in the lower percentiles. This means that children who would not be classified as overweight are still at risk of accumulating more body fat in the future if they show an acceleration in their BMI growth trajectory, also known as excessive gain in BMI z-score.

To be able to screen for infants who are at risk for future overweight, various prediction models were developed using parental, perinatal, and infant characteristics available by the age of 2 years [22–26]. Alongside parental BMI and educational level, measures of infant growth were among the strongest predictors for future overweight and rapid growth [22–26]. These include weight, weight-for-length (WfL), BMI, their sex- and age-adjusted z-scores, their change over time (Δ), their values at the infant BMI peak, and prepeak velocity. However, each study only considered a limited selection of growth measures for the derivation of their risk models. While it is accepted that growth measures in infancy are of added value, it remains unknown which growth measure or combination of growth measures offer the most added value for predicting future overweight and rapid growth in addition to predictors known at birth.

We investigated this by comparing the performances of different combinations of growth measures in infants up to 2 years of age for predicting ΔBMI z-score and excessive gain in BMI z-score between 2 and 5–7 years of age, including the validation thereof in an external cohort.

## Methods

### Study data

Data was obtained from two Dutch population-based birth cohorts: the ABCD (Amsterdam Born Children and their Development) study and the GECKO (Groningen Expert Center for Kids with Obesity) Drenthe study [27, 28]. Data from both cohorts are anonymized and available on request from abcd@amc.nl and www.birthcohorts.net, respectively. Ethical approval for the ABCD study was provided by the Central Committee in Research involving Human Subjects in the Netherlands, the Medical Ethical Committees of participating hospitals, and the Registration Committee of the Municipality of Amsterdam. The GECKO Drenthe study was approved by the Medical Ethics Committee of the University Medical Center Groningen. Informed consent was obtained from all subjects and their legal guardian(s).

The ABCD study was used as the derivation cohort. Between January 2003 and March 2004, all pregnant women were asked to participate at their first visit to an obstetric care provider. 8266 pregnant women in Amsterdam who filled out the pregnancy questionnaire (including sociodemographic characteristics, medical history, and lifestyle), of which 7050 (85%) consented to be followed-up. After birth, the mothers were asked to fill out a questionnaire about their infant’s health, feeding patterns, and behavior. Two weeks after each child’s fifth birthday, the mothers were approached with a follow-up questionnaire and invitation for a health check (anthropometric measurements). The children’s height and weight measurements from the municipality’s Youth Health Care (Jeugdgezondheidszorg) database were combined with data gathered from the health check [29].

The GECKO Drenthe study was used for external validation. Of the 5326 infants born in Drenthe between April 2006 and April 2007, 2997 (56%) of their mothers consented to participate and 2842 (53%) actively participated in the study. Midwives, general practitioners, and gynecologists collected data on the mother during the third trimester of pregnancy and at birth. Anthropometric data after birth was collected during regular check-up visits to the Well Baby Clinics and Youth Health Care, who also distributed the questionnaires for infant’s health, feeding patterns, and behavior.

### Variables

#### Outcomes

Growth measure z-scores were determined according to the World Health Organization Child Growth Standards [30], adjusted for age and sex. The primary outcome of this study was ΔBMI z-score, calculated as the BMI z-score between ages 5 and 7 years minus the BMI z-score at 24 months. Positive values represent positive deviations from the growth curve as set at 24 months of age, and negative values represent children growing towards lower growth curves. Excessive gain in BMI z-score was defined as a ΔBMI z-score > 0.67 standard deviations which corresponds to a quartile increase, a method that has been previously reported [17]. Overweight, including obesity, was defined as a BMI z-score > 1.310 for boys and > 1.244 for girls based on Cole 2012 [19].

### Growth measures

The measures of growth considered for prediction were weight (kilograms), weight-for-length (weight in kilograms divided by height in centimeters), BMI (weight in kilograms divided by height in meters squared), and their age- and sex-dependent z-scores [30]. Specifically, the values of each growth measure at 1, 6, 12, and 24 months of age were used, as well as Δ between the ages 1–6, 6–24, 1–12, 12–24, and 1–24 months. Growth measures at the BMI peak – the point at which BMI reaches a maximum value between birth and 2 years of age – were also considered [26]. This included the age at BMI peak (ranging from 1 to 730 days) and prepeak velocity (growth measure at BMI peak minus growth measure at 30 days of age, divided by the age at the BMI peak in months minus 1). A value of zero was assigned to prepeak velocity if the BMI peak occurred before 31 days of age.

### Predictors at birth

Based on the availability of variables across both cohorts and possibility to harmonize them [27, 28], the following perinatal variables were included as potential predictors known at birth: Birthweight, preterm birth (gestational age < 37 weeks), sex (male vs. female), parity, c-section birth (yes vs. no), Western ethnicity (migration background from Europe [excluding Turkey], North America, Oceania, Indonesia, and Japan vs. non-Western), maternal educational level (low vs. medium vs. high International Standard Classification of Education [31]), maternal age, maternal pre-pregnancy BMI, mother diagnosed with diabetes (gestational or pre-existing vs. no), smoking during pregnancy (yes vs. no), and average income in neighbourhood of residence (≤ 20th vs. 20th -80th vs. >80th percentiles).

### Data set preparation

Infants were excluded from the analysis if the outcome measure was unavailable or if there were fewer than three measurement waves after birth in which the growth measures were available. Measurement waves up to 30 months of age were used for deriving trajectories. Missing predictors at birth data were handled using multiple imputations with 10 iterations, performed separately for each cohort [32]. Only one imputed dataset was created as there were no missing values among the outcomes and growth measures.

**Fig. 1:**
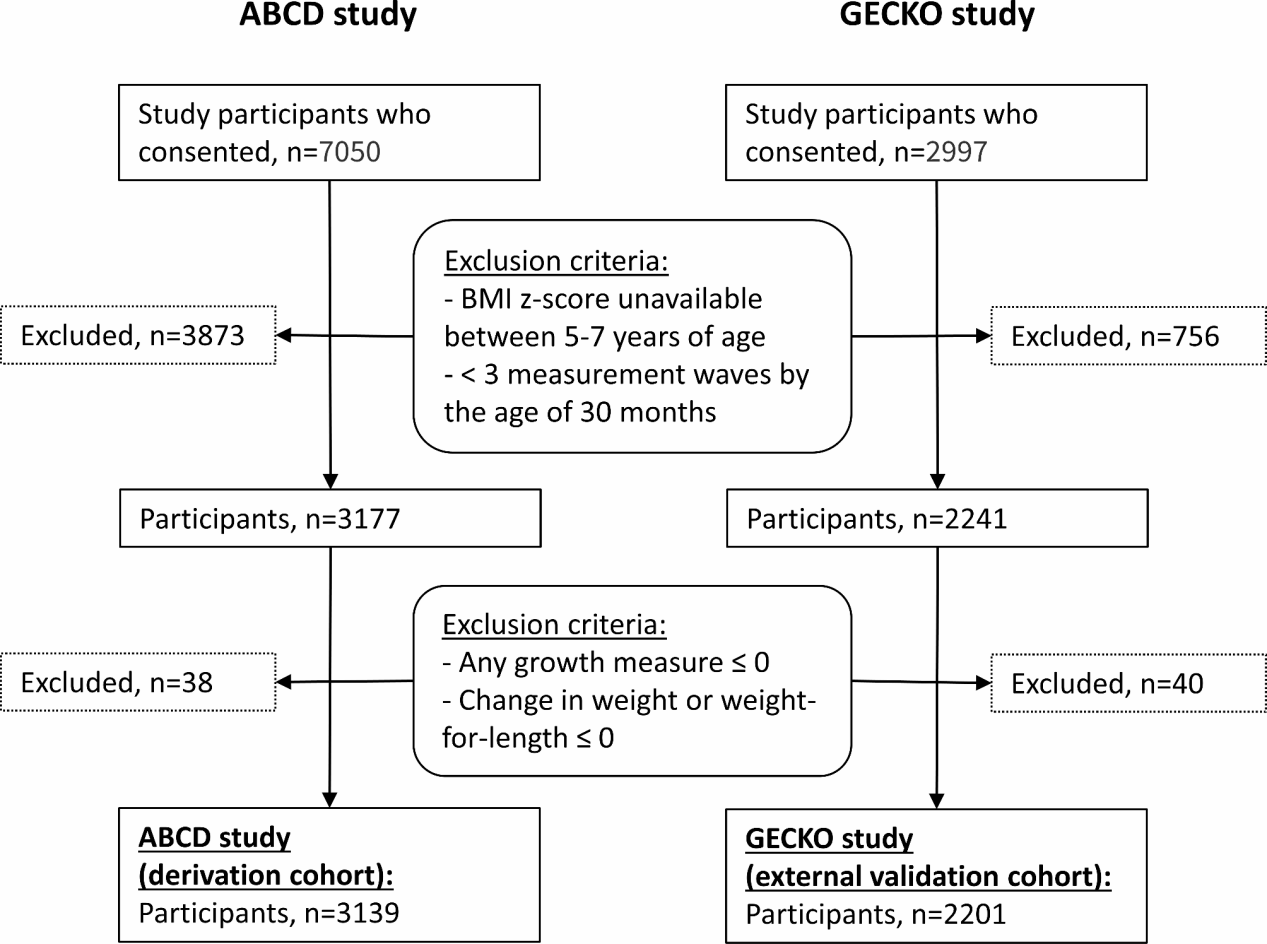
Participant inclusion flowchart

Trajectories of weight and height were derived using linear regression of all data points available to each child, considering fractional polynomial transformations of age up to the fourth degree (selected if the fit was significantly improved [*p* < 0.05]) [33, 34]. WfL and BMI were derived from weight and height at each age of interest. This enabled us to estimate the growth measures at the prespecified ages and at the BMI peak. Infants were excluded from the study if their weight and height trajectories resulted in values less than or equal to zero for weight, WfL, BMI, Δweight change, or ΔWfL (Fig. 1).

### Statistical analysis

All data preparation and analysis were performed in statistical program R version 4.2.0 [35]. Descriptive statistics were reported as means and standard deviations for continuous variables, and frequencies and percentages for categorical variables. All risk prediction models were derived using linear regression considering fractional polynomial transformations of all continuous variables up to the fourth degree (selected if the fit was significantly improved [*p* < 0.01]).

A Birth Model (predictors at birth only) was derived as a reference for model performance of predicting ΔBMI z-score at the time of birth (i.e., no infant growth measures available besides birth weight). 10 models were derived for each of the three growth measures (weight, WfL, and BMI) with the following structures. Model 1: measures at 6, 12, and 24 months. Model 2: Δmeasures at 1–6 months and 6–24 months. Model 3: Δmeasures at 1–12 months and 12–24 months. Model 4: Δmeasures at 1–24 months. Model 5: age and measure at the BMI peak. Model 6: measures at 6, 12, and 24 months + age and measures at the BMI peak. Model 7: Δmeasures at 1–6 months and 6–24 months + age and measures at the BMI peak. Model 8: Δmeasures at 1–12 months and 12–24 months + age and measures at the BMI peak. Model 9: Δmeasures at 1–24 months + age and measures at the BMI peak. Model 10: Δmeasures at 1 month to BMI peak and BMI peak to 24 months + age and measures at the BMI peak.

Finally, two Parsimonious Models were derived with the goal of achieving the best predictive performance using all variables available from conception up to 2- and 1-year(s) of age; these are referred to as the 2-year Parsimonious Model and 1-year Parsimonious Model, respectively. All growth measures and predictors at birth were considered, but variables which did not significantly contribute to the model (p > 0.05 or residual standard deviation [RSD] reduction < 0.005) were removed using backward stepwise regression.

Model performances were reported as RSD (i.e., standard deviation of the difference between the true and predicted ΔBMI z-scores, where a higher value indicates a poorer estimate) and area under the receiver operating characteristic curve (AUC) for predicting excessive gain in BMI z-score (where a higher value indicates a better discriminatory performance). AUC confidence intervals were calculated using the DeLong method [36]. Test characteristics (i.e., true positives, false positives, true negatives, false negatives, sensitivity, specificity, positive predictive value, and negative predictive value) for predicting excessive gain in BMI z-score were reported at a fixed sensitivity value of 0.275 (i.e., using different ΔBMI z-score thresholds per model). Additionally, the models’ Akaike Information Criterion and adjusted R^2^ were reported (Tables S4,S5).

Validation was performed by taking each model’s coefficients (i.e., derived from the derivation cohort) to calculate the risk score of each participant from the validation cohort. Calibration of each model was performed using linear regression – with the validation cohort’s risk scores as the sole predictor for BMI z-score – and applying the newly derived intercept and coefficient to the risk scores.

The described statistical methods were also used to derive models for predicting BMI z-score and overweight at 5–7 years, for which the results are reported in Tables S4-S10.

## Results

The derivation cohort consisted of 3139 infants from the ABCD study, and the external validation cohort of 2201 infants from the GECKO study (Fig. 1). Both ABCD and GECKO infants had an average of 9 measurement waves by 30 months of age which were used to derive height and weight trajectories. The descriptive statistics for each cohort before and after imputations are given in Tables S1 and 1, respectively. Compared to the derivation cohort, infants in the validation cohort were more likely to be overweight at 5–7 years (ABCD = 13.0% vs. GECKO = 22.9%), have an excessive gain in BMI z-score between 2 and 5–7 years (11.9% vs. 26.9%), be both overweight and have an excessive gain in BMI z-score (3.2% vs. 8.4%), and be delivered via c-Sect. (12.0% vs. 15.7%); their mothers were more likely to be of Western ethnicity (66.9% vs. 97.6%), be less educated (17.6% vs. 35.8%), and have smoked during pregnancy (7.9% vs. 14.9%).

Table 1 summarizes the performance across all models in the derivation and external validation cohorts. Overall, a lower RSD for estimating the ΔBMI z-score corresponded with a higher AUC for discriminating between infants (indicating better accuracy and discriminatory performance, respectively).

**Table 1:** Performance of models at predicting ΔBMI z-score and excessive gain in BMI z-score between 2 and 5–7 years of age for each infant growth measure

Cohort	Model	ΔBMI z-score residual standard deviation			AUC (95% CI) for predicting excessive gain in BMI z-score		
		Weight, kg	WfL, kg/cm	BMI, kg/m^2^	Weight, kg	WfL, kg/cm	BMI, kg/m^2^
ABCD (derivation cohort)	Birth Model	0.902			0.690 (0.662–0.717)		
	Model 1	0.867	0.856	0.724	0.734 (0.707–0.760)	0.662 (0.634–0.689)	**0.855 (0.835–0.876)**
	Model 2	0.872	0.872	0.800	0.720 (0.693–0.748)	0.743 (0.717–0.770)	0.795 (0.771–0.819)
	Model 3	0.873	0.872	0.802	0.720 (0.693–0.748)	0.716 (0.689–0.743)	0.795 (0.771–0.819)
	Model 4	0.885	0.876	0.874	0.706 (0.678–0.733)	0.715 (0.688–0.742)	0.728 (0.702–0.755)
	Model 5	0.818	0.821	0.765	0.787 (0.763–0.812)	0.709 (0.682–0.737)	0.827 (0.805–0.849)
	Model 6	**0.808**	**0.799**	**0.722**	**0.796 (0.773–0.820)**	0.787 (0.762–0.811)	**0.855 (0.834–0.876)**
	Model 7	0.810	0.803	0.729	0.793 (0.769–0.817)	**0.802 (0.779–0.825)**	0.851 (0.830–0.872)
	Model 8	0.810	0.806	0.731	0.793 (0.769–0.817)	0.799 (0.775–0.822)	0.852 (0.832–0.873)
	Model 9	0.813	0.810	0.743	0.789 (0.765–0.813)	0.796 (0.772–0.819)	0.844 (0.823–0.865)
	Model 10	0.834	0.833	0.731	0.765 (0.741–0.789)	0.791 (0.768–0.815)	**0.855 (0.835–0.876)**
GECKO (external validation cohort)	Birth Model	0.935			0.491 (0.464–0.518)		
	Model 1	0.906	0.907	0.740	0.563 (0.535–0.591)	0.572 (0.544-0.600)	0.774 (0.751–0.798)
	Model 2	0.916	0.931	0.824	0.558 (0.529–0.586)	0.538 (0.510–0.566)	0.694 (0.668–0.719)
	Model 3	0.919	0.931	0.830	0.552 (0.524–0.581)	0.536 (0.508–0.564)	0.692 (0.667–0.718)
	Model 4	0.940	0.938	0.916	0.517 (0.489–0.545)	0.534 (0.506–0.562)	0.555 (0.528–0.583)
	Model 5	0.823	0.840	0.787	0.669 (0.644–0.695)	0.649 (0.623–0.675)	0.729 (0.704–0.753)
	Model 6	**0.814**	**0.821**	**0.738**	**0.681 (0.656–0.706)**	**0.676 (0.651–0.701)**	**0.776 (0.753–0.799)**
	Model 7	0.820	0.854	0.760	0.676 (0.650–0.701)	0.666 (0.641–0.692)	0.763 (0.739–0.787)
	Model 8	0.819	0.837	0.747	0.677 (0.652–0.703)	0.666 (0.640–0.691)	0.769 (0.745–0.792)
	Model 9	0.827	0.850	0.762	0.666 (0.640–0.691)	0.649 (0.624–0.675)	0.756 (0.732–0.780)
	Model 10	0.867	0.868	0.739	0.623 (0.597–0.649)	0.621 (0.595–0.647)	0.774 (0.751–0.797)

Model 1: Absolute measures at 6, 12, and 24 months. Model 2: Difference between measures at 1–6 months and 6–24 months. Model 3: Difference between measures at 1–12 months and 12–24 months. Model 4: Difference between measures at 1–24 months. Model 5: age and measure at the BMI peak. Model 6: Absolute measures at 6, 12, and 24 months + age and measures at the BMI peak. Model 7: Difference between measures at 1–6 months and 6–24 months + age and measures at the BMI peak. Model 8: Difference between measures at 1–12 months and 12–24 months + age and measures at the BMI peak. Model 9: Difference between measures at 1–24 months + age and measures at the BMI peak. Model 10: Difference between measures at 1 month to BMI peak and BMI peak to 24 months + age and measures at the BMI peak

The best performancein each column and cohort is boldfaced (multiple in the case of a tie)

CI, confidence intervals; AUC, area under the receiver operating characteristic curve; BMI, body mass index, WfL, weight-for-length

In the derivation cohort, the performance parameters of the Birth Model were RSD = 0.902 and AUC = 0.690). The performance was increased when growth measure differences between time points excluding measures at the BMI peak (Models 2–4) were considered (RSD range = 0.800-0.885, AUC range = 0.706–0.795). Combining growth measures with measures at the BMI peak (Models 6–10) achieved better performances overall (RSD range = 0.722–0.834, AUC range = 0.765–0.855). Considering measures at predefined ages (Model 1) and exclusively measures at the BMI peak (Model 5) achieved relatively poor performances when using measures of weight and WfL (RSD range = 0.818–0.867, AUC range = 0.662–0.787), but a better performance when using BMI (RSD range = 0.724–0.765, AUC = 0.827–0.855).

The overall performance in the external validation cohort was worse than in the derivation cohort. On average, the RSD was 0.031 higher and the AUC was 0.126 lower in the validation cohort. Otherwise, the performance between models within the validation cohort showed similar trends as in the derivation cohort. Model calibration on the validation cohort resulted in a mean RSD reduction of 0.036 (Table S3).

When comparing the 3 growth measures across cohorts, models using BMI performed best for predicting both ΔBMI z-score and excessive gain in BMI z-score. The performance between models using weight and WfL were comparable, as well as between weight and weight z-score models, and BMI and BMI z-score models (Table 1,S2). However, WfL z-score models performed better than WfL models based on both metrics.

The coefficients for the 2-year and 1-year Parsimonious Models are reported in Table 2, and their performance measures in Table 3. For both models, the best predictors at birth for ΔBMI z-score were birthweight, sex, parity, educational level, ethnicity, pre-pregnancy BMI, diabetes diagnosis, and smoking during pregnancy. In the 2-year Parsimonious Model, the included growth measures were 24-month weight, 24-month WfL, 24-month BMI, and 12-month BMI. In the 1-year Parsimonious Model, these were weight, WfL, and BMI at 6 and 12 months, BMI at the BMI peak, and the prepeak velocity. Both models included age at BMI peak.

**Table 2:** Coefficients of two Parsimonious Models for predicting ΔBMI z-score between 2 and 5–7 years of age

	2-year Parsimonious Model		1-year Parsimonious Model	
**Variable**	**Coefficient**	**99% CI**	**Coefficient**	**99% CI**
Intercept	-18.006	-20.523 to -15.489	2.324	1.048, 3.599
(Birthweight/10,000)^−2^, g	-0.068	-0.095 to -0.042	-0.107	-0.138, -0.076
(Birthweight/10,000)^−2^ × $ln$(Birthweight/10,000), g	-0.028	-0.040 to -0.017	-0.043	-0.056, -0.030
Male sex, yes	0.467	0.415 to 0.519	0.434	0.377, 0.490
Parity, count	-0.057	-0.086 to -0.027	-0.059	-0.090, -0.029
Middle maternal educational level, yes	-0.097	-0.185 to -0.010	-0.112	-0.203, -0.022
High maternal educational level, yes	-0.150	-0.242 to -0.059	-0.153	-0.248, -0.058
Western ethnicity, yes	-0.115	-0.186 to -0.044	-0.121	-0.194, -0.047
(Pre-pregnancy maternal BMI/10)^3^, kg/m^2^	0.069	0.054 to 0.085	-0.031	-0.043, -0.020
(Pre-pregnancy maternal BMI /10)^3^ × $ln$(Pre-pregnancy maternal BMI /10), kg/m^2^	-0.040	-0.050 to -0.030	-	-
(Pre-pregnancy maternal BMI/10)^2^, kg/m^2^	-	-	0.191	0.139, 0.243
Mother diagnosed with diabetes, yes	0.158	-0.026 to 0.341	0.158	-0.026, 0.342
Smoking during pregnancy, yes	0.153	0.048 to 0.257	0.148	0.040, 0.256
(Weight at 24 months/10,000)^−1^, grams	2.589	0.816 to 4.363	-	-
(Weight at 24 months /10,000)^3^, grams	0.405	0.276 to 0.535	-	-
$ln$(WfL at 24 months/0.1), kg/cm	9.183	6.152 to 12.214	-	-
$ln$(WfL at 24 months/0.1)^2^, kg/cm	-11.034	-13.959 to -8.109	-	-
BMI at 12 months/10, kg/m^2^	2.131	1.757 to 2.505	-	-
(BMI at 24 months/10)^−1^, kg/m^2^	14.926	13.985 to 15.867	-	-
Weight at 6 months/1000, kg	-	-	-1.359	-1.706, -1.011
Weight at 12 months/1000, kg	-	-	1.375	1.114, 1.636
WfL at 6 months/0.1, kg/cm	-	-	4.416	1.678, 7.153
WfL at 12 months/0.1, kg/cm	-	-	-8.320	-10.560, -6.081
BMI at 6 months, kg/m^2^	-	-	1.094	0.961, 1.228
BMI at 12 months, kg/m^2^	-	-	-1.111	-1.251, -0.971
BMI at BMI peak/10, kg/m^2^	-	-	-0.898	-1.246, -0.549
((Prepeak velocity + 0.1)/0.1)^−1^, kg/m^2^/month	-	-	-0.664	-0.891, -0.438
(Prepeak velocity + 0.1)/0.1)^−1^ × $ln$((Prepeak velocity + 0.1)/0.1), kg/m^2^/month	-	-	-3.582	-4.530, -2.635
(Age at BMI peak/100)^−0.5^, days	-	-	-1.750	-2.364, -1.137
(Age at BMI peak/100)^−0.5^ × $ln$(Age at BMI peak), days	-	-	-0.432	-0.573, -0.291
Age at BMI peak between 2 and 365 days, yes	-0.115	-0.223 to -0.007	4.532	3.095, 5.969
Age at BMI peak > 365 days, yes	-0.445	-0.869 to -0.020	4.522	3.091, 5.953

Standard errors are heteroskedasticity robust. The equation for estimating an infant’s ΔBMI z-score between 2 and 5–7 years of age is ${\beta }_{0}+{\beta }_{1}{x}_{1}+{\beta }_{2}{x}_{2}+\dots {\beta }_{n}{x}_{n}$, where ${\beta }_{0}$ is the intercept’s coefficient, ${\beta }_{1}$ to ${\beta }_{n}$ are the coefficients for each variable in the model (where $n$ is the total number of variables), and ${x}_{1}$ to ${x}_{n}$ are the infant’s values for each variable (for continuous variables, insert the corresponding value in the correct units; for categorical variables, insert 1 if the factor is present and 0 if not)

BMI, body mass index; CI, confidence interval, WfL, weight-for-length

**Table 3:** Performance of the two Parsimonious Models and the Birth Model for predicting excessive gain in BMI z-score between 2 and 5–7 years of age at a fixed sensitivity threshold of at 0.275

	2-year Parsimonious Model		1-year Parsimonious Model		Birth Model	
Performance measure	ABCD	GECKO	ABCD	GECKO	ABCD	GECKO
Residual standard deviation	0.714	0.741	0.735	0.739	0.902	0.935
AUC (95% CI)	0.856 (0.835–0.876)	0.766 (0.743–0.790)	0.846 (0.825–0.867)	0.759 (0.735–0.783)	0.690 (0.662–0.717)	0.491 (0.464–0.518)
ΔBMI z-score threshold	0.69	0.55	0.56	0.51	0.67	-0.15
True positives (%)	102 (71.8)	163 (79.9)	104 (60.8)	163 (71.8)	123 (30.0)	162 (25.4)
True negatives (%)	2726 (91.0)	1568 (78.5)	2699 (90.9)	1545 (78.3)	2406 (88.2)	1134 (72.5)
False positives (%)	40 (28.2)	41 (20.1)	67 (39.2)	64 (28.2)	287 (70.0)	475 (74.6)
False negatives (%)	271 (9.0)	429 (21.5)	269 (9.1)	429 (21.7)	323 (11.8)	430 (27.5)
Sensitivity	0.273	0.275	0.279	0.275	0.276	0.274
Specificity	0.986	0.975	0.976	0.960	0.893	0.705
Positive predictive value	0.718	0.799	0.608	0.718	0.300	0.254
Negative predictive value	0.910	0.785	0.909	0.783	0.882	0.725

AUC, area under the receiver operating characteristic curve; BMI, body mass index; CI, confidence interval

The 2-year Parsimonious Model was minimally superior to the 1-year Parsimonious Model for estimating ΔBMI z-score (Table 3). At a fixed sensitivity, both Parsimonious Models achieved higher specificities, positive predictive values, and negative predictive values than the Birth Model for predicting excessive gain in BMI z-score. Given a positive test result (i.e., excessive gain in BMI z-score predicted), the proportion of false positives for the 2-year Parsimonious Model, 1-year Parsimonious Model, and Birth Model were 28.2%, 39.2%, and 70.0%, respectively, in the derivation cohort and 20.1%, 28.2%, and 74.6%, respectively, in the validation cohort. The respective percentages of false negative predictions out of all negative results were 9.0%, 9.1%, and 11.8% in the derivation cohort and 21.5%, 21.7%, and 27.5% in the validation cohort. Decision curve analyses for the Parsimonious Models and Birth Model in the external validation cohort are portrayed in Figure S1, including how it can aid clinical decision making [37, 38].

**Fig. 2:**
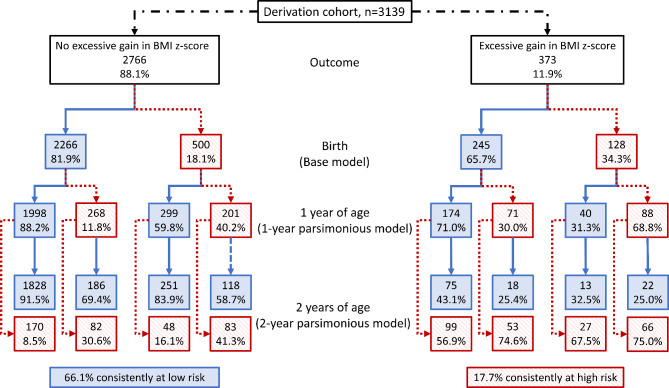
Categorization flowchart of children in the derivation cohort as high (> 80th risk percentile, given in red boxes) vs. low risk (≤ 80th risk percentile, given in blue boxes) at birth, 1 year of age, and 2 years of age using the Birth Model, 1-year Parsimonious Model, and 2-year Parsimonious Model, respectively, stratified by excessive gain in BMI z-score (> 0.67) between 2 and 5–7 years of age or not. BMI, body mass index

**Fig. 3:**
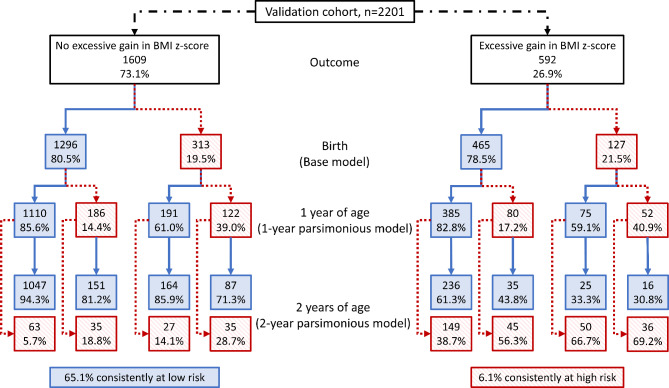
Categorization flowchart of children in the validation cohort as high (> 80th risk percentile, given in red boxes) vs. low risk (≤ 80th risk percentile, given in blue boxes) at birth, 1 year of age, and 2 years of age using the Birth Model, 1-year Parsimonious Model, and 2-year Parsimonious Model, respectively, stratified by excessive gain in BMI z-score (> 0.67) between 2 and 5–7 years of age or not. BMI, body mass index

Figures 2 and 3 summarize a hypothetical scenario where children in the derivation and validation cohorts, respectively, with a risk score above the 80% percentile would be categorized as high risk. The figures show the flowchart of high and low risk categorization across 3 measurement timepoints: at birth, 1 year of age, and 2 years of age. In the derivation cohort, 66.1% of children who did not experience excessive gain in BMI z-score were consistently categorized as low risk, while 17.7% with excessive gain in BMI z-score were consistently categorized as high risk. The respective proportions in the validation cohort were 65.1% and 6.1%. Using this strategy, 96.1% ($\frac{1828}{1828+75}$) of infants consistently at low risk would not encounter excessive gain in BMI z-score in the derivation cohort; the proportion in the validation cohort is 81.6% ($\frac{1047}{1047+236}$). Conversely, 44.3% ($\frac{66}{83+66}$) and 50.7% ($\frac{36}{35+36}$) of infants consistently at high risk would later experience excessive gain in BMI z-score in the derivation and validation cohorts, respectively.

Similar trends and performances were found for the risk models for predicting BMI z-score and overweight at 5–7 years of age (Tables S4-S10).

## Discussion

The motivation for our study was to criticize published risk models for predicting growth and weight in children, and how their selection of growth measures and model structures do not appear to have been sufficiently explored in search of optimal performance. We hereby compared different growth measures up to 2 years of age – namely weight, WfL, and BMI – and their ability to predict ΔBMI z-score and excessive gain in BMI z-score between the ages of 2 and 5–7 years. Ten model structures were assessed for each growth measure, varying in the use of absolute measures, the difference in measures between two timepoints, and measures related to the BMI peak.

We found that predictors at birth – related to the mother, pregnancy, and delivery – have a weak predictive power, and that the addition of any growth measures improves model performance. As the predictors at birth remained constant across the models, this means that risk groups could be identified at birth and updated at later timepoints with the addition of growth measures. Using BMI as a growth measure led to better ΔBMI z-score estimates and better discrimination between children with and without excessive gain in BMI z-score. Despite both cohorts being based in Dutch cities, most models performed less well in the validation cohort. This is likely a consequence of having used data-driven variable selection methods without regard for causality. Models whose purpose are to translate well to external datasets should hereby consider relationships and dependencies between variables and actively prevent overfitting and multicollinearity.

When all growth measures were considered for the Parsimonious Models, 3 growth measures included in the 2-year Parsimonious Model were weight, WfL, and BMI at 24 months. This indicates that information on body size available from the latest measurement wave is likely to best predict future body size [16, 25]. However, the growth trajectory is likely still to be of some added value given that BMI at 12 months was also included in the model. The growth measures included in the 1-year Parsimonious Model were weight, WfL, and BMI at 6 and 12 months. This suggests that the growth trajectory offers a greater role as a predictor for ΔBMI z-score in infants younger than 2 years.

It is noteworthy that the prevalence of overweight and excessive gain in BMI z-score between 2 and 5–7 years of age in the validation cohort was approximately double that of the derivation cohort, while the distribution of growth measures between birth and 24 months was equivalent between the cohorts (Table 4). This means that growth measures beyond 24 months of age discriminate future weight status better than growth measures up to 24 months [16], and the growth pattern in infancy is predictive of future growth rate [9, 14, 15]. This is supported by the fact that both Parsimonious Models included measures from multiple measurement waves.

**Table 4:** Descriptive statistics after imputations

Variables	ABCD cohort, n = 3139	GECKO cohort, n = 2201
BMI z-score at 5–7 years (SD)	-0.04 (0.96)	0.43 (0.92)
Overweight at 5–7 years (%)	264 (8.4)	330 (15.0)
ΔBMI z-score 2 to 5–7 years (SD)	-0.39 (0.95)	0.19 (0.94)
Excessive gain in BMI z-score 2 to 5–7 years (%)	373 (11.9)	592 (26.9)
Weight at 1 month, kg (SD)*	5.3 (0.9)	5.1 (0.9)
Weight at 6 months, kg (SD)*	7.4 (0.8)	7.5 (0.8)
Weight at 12 months, kg (SD*	9.5 (1.1)	9.7 (1.1)
Weight at 24 months, kg (SD)*	13.6 (1.9)	13.8 (2.1)
Weight-for-length at 1 month, kg/cm (SD)*	0.09 (0.01)	0.09 (0.01)
Weight-for-length at 6 months, kg/cm (SD)*	0.11 (0.01)	0.11 (0.01)
Weight-for-length at 12 months, kg/cm (SD)*	0.13 (0.01)	0.13 (0.01)
Weight-for-length at 24 months, kg/cm (SD)*	0.16 (0.02)	0.16 (0.02)
BMI at 1 month, kg/m^2^ (SD)*	15.7 (1.9)	15.5 (2.1)
BMI at 6 months, kg/m^2^ (SD)*	17.0 (1.3)	17.0 (1.3)
BMI at 12 months (k, m^2^ (SD)*	17.2 (1.3)	17.1 (1.3)
BMI at 24 months, kg/m^2^ (SD)*	16.4 (1.4)	16.3 (1.4)
Age at BMI peak, months (SD)*	10.4 (5.0)	9.3 (4.9)
Weight at BMI peak, kg (SD)*	5.4 (0.9)	5.3 (1.0)
Weight-for-length at BMI peak, kg/cm (SD)*	0.09 (0.01)	0.09 (0.01)
BMI at BMI peak, kg/m^2^ (SD)*	17.6 (1.7)	17.7 (1.9)
Prepeak velocity, kg/m^2^/month (SD)*	0.2 (0.3)	0.2 (0.4)
Birthweight, kg (SD)	3.5 (0.5)	3.5 (0.6)
Preterm birth, (%)	150 (4.8)	110 (5.0)
Female sex (%)	1587 (50.6)	1099 (49.9)
Parity (%)	0.7 (0.9)	0.8 (0.8)
C-section delivery (%)	377 (12.0)	346 (15.7)
Western ethnicity (%)	2099 (66.9)	2149 (97.6)
Mother’s educational level	Reference	Reference
Low (%)	552 (17.6)	787 (35.8)
Medium (%)	1024 (32.6)	649 (29.5)
High (%)	1563 (49.8)	765 (34.8)
Mother’s age, years (SD)	31.2 (5.3)	31.3 (4.4)
Mother’s pre-pregnancy BMI (%)	23.3 (4.2)	24.8 (4.9)
Mother diagnosed with diabetes (%)	88 (2.8)	79 (3.6)
Smoking during pregnancy (%)	247 (7.9)	329 (14.9)
Neighbourhood income percentile	Reference	Reference
≤ 20th percentile (%)	627 (20.0)	437 (19.9)
20th-80th percentile (%)	1866 (59.4)	1312 (59.6)
> 80th percentile (%)	646 (20.6)	452 (20.5)

Continuous variables are given in means with standard deviations in brackets; categorical variables are given in frequencies with percentages in brackets. In the ABCD cohort, the number of missing values imputed were: preterm birth = 5, c-section delivery = 350, Western ethnicity = 6, mother’s educational level = 28, smoking during pregnancy = 92. In the GECKO cohort, this was: preterm birth = 14, parity = 9, c-section delivery = 196, Western ethnicity = 119, mother’s educational level = 122, mother’s age = 4, mother’s pre-pregnancy BMI 149, mother diagnosed with diabetes = 12, smoking during pregnancy = 8, neighbourhood income percentile = 204

BMI, body mass index; SD, standard deviation

*Based on modelled height and weight trajectories

Another consideration for the application of risk prediction models in practice are the benefits of correctly predicting excessive gain in BMI z-score (true positives) and the drawbacks of misprediction (false positives). Based on the performance of the 2-year Parsimonious Model at the 0.275 sensitivity threshold in the validation cohort, only 2.5% of infants who will not experience excessive gain in BMI z-score would receive a positive test (1-specificity) and 79.9% of infants with a positive test would later experience an excessive gain in BMI z-score (positive predictive value). However, there is considerable variability in growth pattern across infants. Using a multi-timepoint risk assessment strategy similar to the categorization flowcharts may provide a guideline to personalize monitoring among infants [25, 39]. Given that most children will not experience excessive gain in BMI z-score and that it is easier to correctly predict this outcome, an efficient strategy might be increasing the monitoring among high-risk infants and reduce monitoring among those at lower risk.

Other models have attempted to predict overweight or obesity at approximately 2 years of age [40, 41]. Given the rapid growth and inaccurate height measurement before the age of 2 years, small changes in reported values can lead to large differences in BMI compared to weight or WfL (height is squared in the BMI equation). BMI in infancy was therefore considered to be unreliable and weakly associated with adolescent and adult obesity [42, 43]. However, these models only incorporated three or four measurement waves. We included an average of 9 measurement waves to derive our trajectories, which resulted in the finding that BMI was usually the best growth measure for predicting future BMI z-score. This implies that non-linear BMI trajectories are only unreliable when few measurement waves are available, whereas a larger number of measurements result in growth measures with stronger predictive abilities.

Though not the focus of our study, we found that using the weight z-score and BMI z-score as predictors achieved similar model performances to using weight and BMI as predictors, respectively. An exception was that the WfL z-score resulted in consistently superior models compared to using WfL. Additionally, we also derived models to predict BMI z-score at 5–7 years (Tables S4-S10). Similar trends were found between the respective models for predicting BMI z-score (and overweight) vs. ΔBMI z-score (and excessive gain in BMI z-score). Note that there is only moderate overlap between children with overweight and children with excessive gain in BMI z-score. This suggests that the association of rapid growth towards overweight is either not always apparent between 2 and 5–7 years of age and depends on whether a child follows a high or low growth trajectory. Given that early rapid growth has a stronger association with negative health outcomes later in life [7–18], screening should likely focus on ΔBMI z-score.

Zhang et al. [44] also derived various risk models for predicting overweight in children based on multiple growth measures up to the age of 2 years (i.e., height z-score, weight z-score, Δweight z-score, BMI, and BMI z-score). Their focus was to compare different data mining methods, so all growth measures were included in each risk model. Their conclusion was that Bayesian algorithms and support vector machines achieved the best performing models and logistic regression models were among the worst performing. However, AUC was not reported and thresholds were selected based on Youden’s index. The results are uninformative because, firstly, sensitivity and specificity are inversely related; one must be fixed to be able to use the other as a performance measure. Secondly, accuracy is biased towards the most prevalent outcome (i.e., not overweight participants), so relatively small decreases in accuracy and specificity correspond to larger increases in sensitivity.

### Strengths and limitations

The main strengths of our study were the availability of many measurement waves within the first 2.5 years of age – which enabled more accurate individual growth trajectory estimations – and the use of an external cohort with different demographics for validation. Additionally, we included multiple growth measures and reported the results from total of 126 models for predicting ΔBMI z-score between 2 and 5–7 years and BMI z-score at 5–7 years across 6 growth measures.

A limitation is that the measures of infant growth were independently extracted from individual’s modelled weight and height trajectories. Although this was necessary to estimate growth measures at specific ages for the models, the accuracy of the trajectories is difficult to validate. 38/3177 (1%) infants from the derivation cohort and 40/2241 (2%) from the validation cohort were excluded due to implausible estimates. It is likely that the measures at the trajectory tails (i.e., at 1 and 24 months of age) are less accurate, especially for participants who may lack measurement waves near the tails. To compensate, we included measurement timepoints up to 30 months in the trajectory models to improve the estimates at 24 months. The fact that 9 measurement waves were available on average greatly improves the trajectory reliability compared to the minimum requirement of 3 measurements.

We acknowledge that the model structures considered can be considered arbitrary as many other possibilities exist. However, we attempted to be thorough while avoiding an overwhelming number of models. We also note that all growth measures are highly correlated with each other, meaning that different measures may have been included into the Parsimonious Models with minor methodological alterations. Within the confines of this study, we acknowledge that our attempt to causally interpret the growth measures selected for the Parsimonious Models are mostly speculative.

## Conclusion

It is possible, with moderate accuracy, to predict ΔBMI z-score and excessive gain in BMI z-score between 2 and 5–7 years of age based on growth measures within the first 2 years of age. There is a clear improvement in predictive power when growth trajectories are included as predictors in addition to predictors at birth. Growth measures at the most recently available measurement wave (i.e., 2 years) seem to best predict ΔBMI z-score, with a greater added value of the past growth trajectories for predictions at an earlier age (i.e., 1 year). BMI was an overall better predictor compared to weight or WfL.

The pros and cons of using risk prediction models to guide public health interventions should be carefully assessed before their implementation in practice. Regardless, our study endorses the monitoring of growth trajectories from infancy, with the identification of rapid growth to be considered as a risk factor for future overweight and related cardiometabolic diseases. We have demonstrated that many risk models can be derived for such predictions, and that the performance across models can differ significantly. The process of developing an optimal model for widespread applications hereby necessitates extensive internal and external testing of the many possibilities.

### Electronic supplementary material

Below is the link to the electronic supplementary material.

Supplementary Material 1

## Data Availability

The individual ABCD study and GECKO Drenthe study data are not available for a public repository for ethical reasons but can be made available to other researchers upon reasonable request. This can be done by contacting the project leaders of the ABCD study (abcd@amsterdamumc.nl) and GECKO Drenthe study (gecko@tcc.umcg.nl).
