# Supplementary material for: Modelling individual infancy growth trajectories to predict excessive gain in BMI z-score: a comparison of growth measures in the ABCD and GECKO Drenthe cohorts
Source: BMC Public Health. 2023 Dec 5;23:2428. doi: 10.1186/s12889-023-17354-4 (PMC10698894; doi:10.1186/s12889-023-17354-4)
Supplement: Supplementary file 1 — Supplementary Material 1 [file 12889_2023_17354_MOESM1_ESM.docx]

# Modelling individual infancy growth trajectories to predict excessive gain in BMI z-score: a comparison of growth measures in the ABCD and GECKO Drenthe cohorts

Anton Schreuder^1,2,§^, Eva Corpeleijn^3^, Tanja Vrijkotte^1^

^1^ Department of Public and Occupational Health, Amsterdam Public Health Research Institute, Amsterdam UMC, University of Amsterdam, Amsterdam, The Netherlands

^2^ Leiden Institute of Advanced Computer Science, Leiden University, Leiden, The Netherlands

^3^ University of Groningen, University Medical Center Groningen, Department of Epidemiology, Groningen, The Netherlands

^§^ Corresponding author: [antoniusschreuder@gmail.com](mailto:antoniusschreuder@gmail.com)

# Supplement

Contents

[Supplement 2](#_Toc151030523)

[Figure S1: Decision curve analysis for predicting excessive gain in BMI z-score in the external validation cohort 3](#_Toc151030524)

[Table S1: Descriptive statistics of variables with at least one missing case before imputations 5](#_Toc151030525)

[Table S2: Performance of models at predicting ΔBMI z-score and excessive gain in BMI z-score between 2 and 5-7 years of age for each infant z-score growth measure 6](#_Toc151030526)

[Table S3: Residual standard deviation of GECKO-calibrated models at predicting ΔBMI z-score between 2 and 5-7 years of age for each measure of infant growth 8](#_Toc151030527)

[Table S4: Akaike Information Criterion of models at predicting ΔBMI z-score between 2 and 5-7 years of age for each measure of infant growth 9](#_Toc151030528)

[Table S5: Adjusted R^2^ of models at predicting ΔBMI z-score between 2 and 5-7 years of age for each measure of infant growth 10](#_Toc151030529)

[Table S6: Performance of models at predicting BMI z-score and overweight at 5-7 years of age for each infant growth measure 11](#_Toc151030530)

[Table S7: Performance of models at predicting BMI z-score and overweight at 5-7 years of age for each infant z-score growth measure 12](#_Toc151030531)

[Table S8: Residual standard deviation of GECKO-calibrated models at predicting BMI z-score at 5-7 years of age for each measure of infant growth 13](#_Toc151030532)

[Table S9: Coefficients of two Parsimonious risk models for predicting BMI z-score at 5-7 years of age. 14](#_Toc151030533)

[Table S10: Performance of the two Parsimonious Models and the Birth Model for predicting overweight at 5-7 years of age at a fixed sensitivity threshold of at 0.275 15](#_Toc151030534)


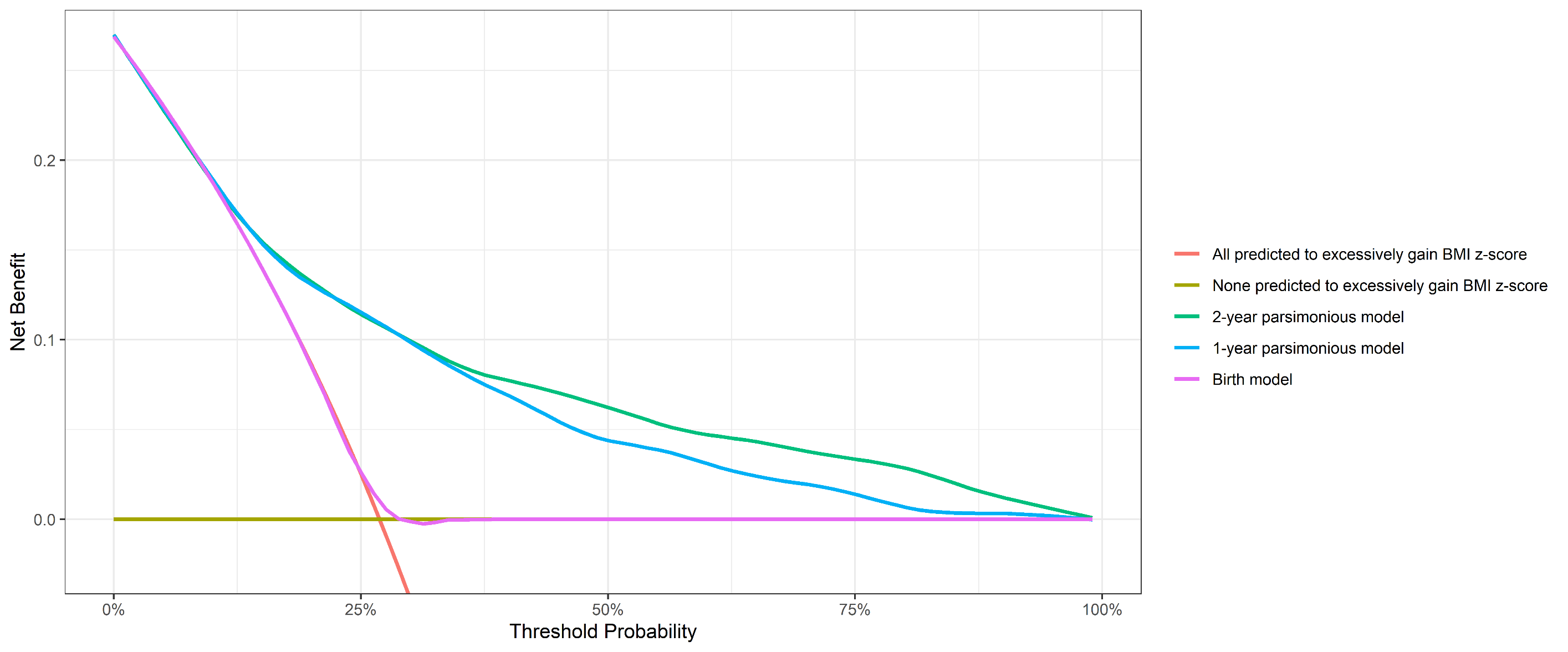

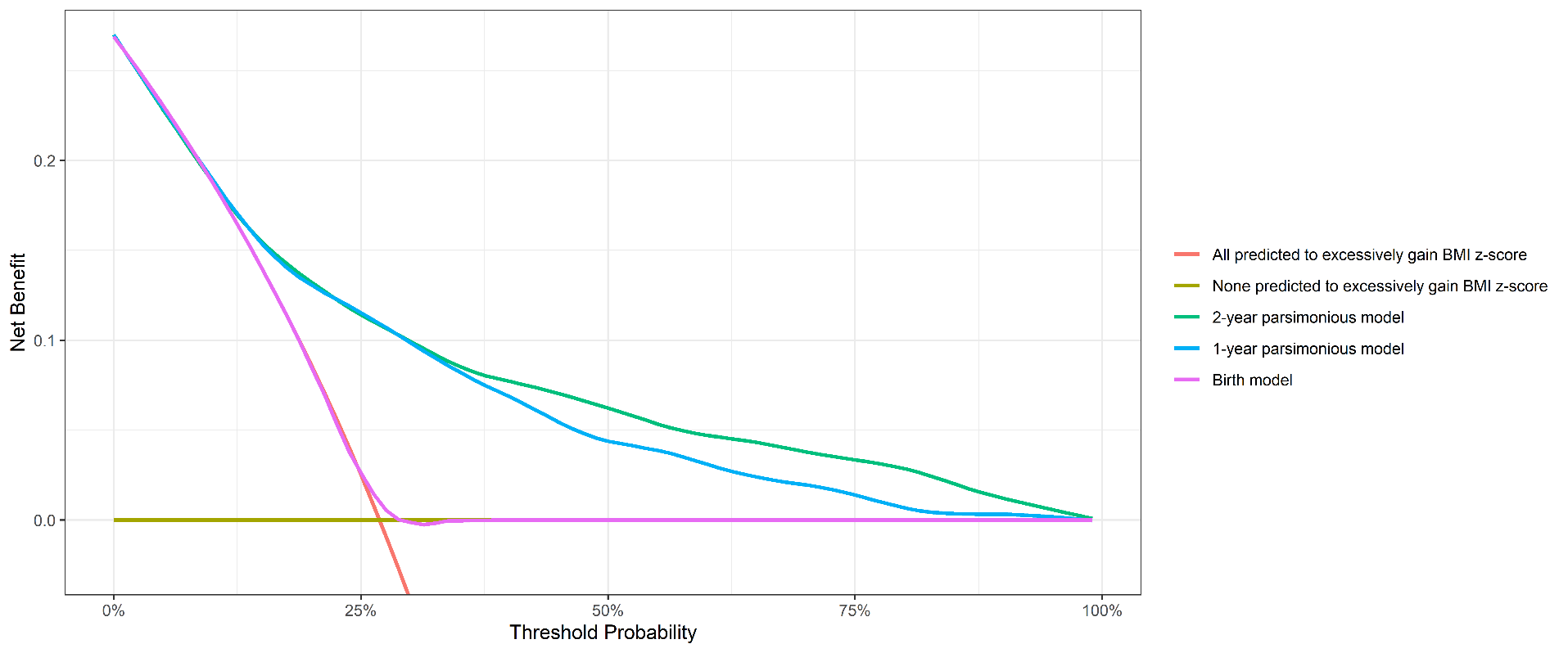


### Figure S1: Decision curve analysis for predicting excessive gain in BMI z-score in the external validation cohort

Decision curve analysis is an aid for clinical decision making: given a test result, which patients should receive the assigned intervention? In this case, it provides an indication whether there is an added benefit to utilizing our models compared to either predicting that all children will excessively gain BMI z-score (i.e., all children should undergo a preventative intervention) or that none of the children will excessively gain BMI z-score (i.e., nobody should undergo a preventative intervention). The risk probability lies between 0% and 100%; any threshold within that range may be selected (i.e., children with a risk score above the threshold probability will receive an intervention, and those with a risk score below the threshold probability will not receive an intervention). Given the desired threshold probability, the decision curve analysis indicates which strategy would lead to the most benefit for all (i.e., intervention for all, intervention for none, or utilize a model for an intermediate option).

Before referring to the decision curve analysis, the decision maker must first determine their desired threshold probability. In this case, a threshold probability of 10% (1/10) means that 1 out of 10 children with a positive test result will excessively gain BMI z-score (9 of the 10 children will not excessively gain BMI z-score [false positives]). A threshold probability of 90% (9/10) means that 9 out of 10 children with a positive test result will truly excessively gain BMI z-score (1 of the 10 children will not excessively gain BMI z-score [false positives]).

The choice of threshold probability is subjective to the decision maker and unique to each scenario. Consider an example where the intervention for children with a positive test is a family-based weight loss treatment program (FBT). FBT may prevent children from excessively gain BMI z-score, so it is beneficial for true positive cases. On the other hand, it would be a waste of time and resources to prescribe FBT to families whose child would not excessively gain BMI z-score. Ultimately, it must be decided on how many true positive cases would justify one false positive case.

At a threshold probability of 25%, this means that it would be justified that 3 families would undergo FBT unnecessarily (false positives) so that 1 family may benefit from FBT (true positive). At a threshold probability of 75%, 1 family would undergo FBT unnecessarily (false positive) for every 3 families who may benefit from FBT (true positives).

After deciding on the preferred threshold probability, draw a vertical line at that value on the decision curve analysis and consider where each line crosses. For example, the net benefit at 25% threshold probability for “All predicted to excessively gain BMI z-score,” “None predicted to excessively gain BMI z-score,” and “2-year Parsimonious Model” would be 0.025, 0, and 0.118, respectively. As a higher value indicates a higher benefit, this means that, at the 25% threshold probability, using the 2-year Parsimonious Model would be more beneficial than assuming that all or none of the children will become overweight. At the threshold probability of <12%, the net benefit for “All predicted to excessively gain BMI z-score” is equal to the ”2-year Parsimonious Model”. This implies that using the model would provide an equivalent benefit to prescribing interventions to all children. This decision curve analysis recommends utilizing the 2-year Parsimonious Model when the threshold probability is above 12%.

The Birth model curve overlaps with the “All predicted to excessively gain BMI z-score” below the 26% threshold probability and with “None predicted to excessively gain BMI z-score” above the 28% threshold probability. The “1-year Parsimonious Model” curve overlaps with the “2-year Parsimonious Model” curve below the 33% threshold probability, above which it results in a lower net benefit.

### Table S1: Descriptive statistics of variables with at least one missing case before imputations

| **Variables** |  | **ABCD cohort, n=3139** |  | **GECKO cohort, n=2201** |
| --- | --- | --- | --- | --- |
|  | **Missing (%)** | **Descriptives** | **Missing (%)** | **Descriptives** |
| Preterm birth, (%) | 5 (0.2) | 149 (4.8) | 14 (0.6) | 109 (5.0) |
| Parity (%) | 0 | 0.7 (0.9) | 9 (0.4) | 0.8 (0.8) |
| C-section delivery (%) | 350 (11.2) | 324 (11.6) | 196 (8.9) | 312 (15.6) |
| Western ethnicity (%) | 6 (0.2) | 2094 (66.8) | 119 (5.4) | 2033 (97.6) |
| Mother's educational level | 28 (0.9) | Reference | 122 (5.5) | Reference |
| Low (%) | - | 537 (17.3) | - | 749 (36.0) |
| Medium (%) | - | 1016 (32.7) | - | 615 (29.6) |
| High (%) | - | 1558 (50.1) | - | 715 (34.4) |
| Mother’s age, years (SD) | 0 | 31.2 (5.3) | 4 (0.2) | 31.3 (4.4) |
| Mother's pre-pregnancy BMI (%) | 0 | 23.3 (4.2) | 149 (6.8) | 24.8 (4.8) |
| Mother diagnosed with diabetes (%) | 0 | 88 (2.8) | 12 (0.5) | 78 (3.6) |
| Smoking during pregnancy (%) | 92 (2.9) | 235 (7.7) | 8 (0.4) | 328 (15.0) |
| Neighbourhood income percentile | 0 | Reference | 204 (9.3) | Reference |
| ≤20th percentile (%) | - | 761 (24.2) | - | 386 (19.3) |
| 20th-80th percentile (%) | - | 1804 (57.5) | - | 1197 (59.9) |
| >80th percentile (%) | - | 574 (18.3) | - | 414 (20.7) |

Continuous variables are given in means with standard deviations in brackets; categorical variables are given in frequencies with percentages in brackets.

BMI, body mass index; SD, standard deviation.

*Based on modelled height and weight trajectories.

### Table S2: Performance of models at predicting ΔBMI z-score and excessive gain in BMI z-score between 2 and 5-7 years of age for each infant z-score growth measure

| **Cohort** | **Model** | **BMI z-score residual standard deviation** | | | **AUC (95% CI) for predicting overweight** | | |
| --- | --- | --- | --- | --- | --- | --- | --- |
|  |  | **Weight z-score, kg** | **WfL z-score, kg/cm** | **BMI z-score, kg/m^2^** | **Weight z-score, kg** | **WfL z-score, kg/cm** | **BMI z-score, kg/m^2^** |
| ABCD (derivation cohort) | Birth model | 0.902 | | | 0.690 (0.662-0.717) | | |
|  | Model 1 | 0.866 | 0.728 | 0.723 | 0.734 (0.707-0.761) | 0.852 (0.831-0.872) | 0.855 (0.834-0.875) |
|  | Model 2 | 0.883 | 0.802 | 0.784 | 0.708 (0.681-0.736) | 0.790 (0.766-0.814) | 0.807 (0.783-0.831) |
|  | Model 3 | 0.883 | 0.790 | 0.783 | 0.706 (0.679-0.734) | 0.801 (0.777-0.825) | 0.809 (0.785-0.834) |
|  | Model 4 | 0.899 | 0.876 | 0.876 | 0.688 (0.660-0.715) | 0.733 (0.706-0.759) | 0.724 (0.696-0.753) |
|  | Model 5 | 0.815 | 0.778 | 0.776 | 0.786 (0.762-0.811) | 0.810 (0.787-0.833) | 0.821 (0.799-0.844) |
|  | Model 6 | 0.804 | 0.727 | 0.722 | 0.796 (0.773-0.820) | 0.851 (0.830-0.872) | 0.854 (0.833-0.875) |
|  | Model 7 | 0.805 | 0.745 | 0.729 | 0.795 (0.771-0.819) | 0.839 (0.818-0.859) | 0.843 (0.821-0.865) |
|  | Model 8 | 0.805 | 0.736 | 0.731 | 0.796 (0.772-0.820) | 0.845 (0.824-0.865) | 0.842 (0.820-0.864) |
|  | Model 9 | 0.810 | 0.754 | 0.749 | 0.789 (0.765-0.813) | 0.834 (0.813-0.855) | 0.827 (0.804-0.850) |
|  | Model 10 | 0.809 | 0.744 | 0.730 | 0.789 (0.765-0.813) | 0.840 (0.819-0.861) | 0.849 (0.829-0.870) |
| GECKO (external validation cohort) | Birth model | 0.935 | | | 0.491 (0.464-0.518) | | |
|  | Model 1 | 0.904 | 0.748 | 0.741 | 0.568 (0.540-0.596) | 0.769 (0.745-0.792) | 0.776 (0.753-0.799) |
|  | Model 2 | 0.923 | 0.830 | 0.803 | 0.548 (0.520-0.577) | 0.692 (0.667-0.718) | 0.714 (0.689-0.739) |
|  | Model 3 | 0.924 | 0.810 | 0.808 | 0.545 (0.517-0.573) | 0.709 (0.683-0.734) | 0.718 (0.693-0.743) |
|  | Model 4 | 0.943 | 0.908 | 0.923 | 0.507 (0.479-0.535) | 0.562 (0.535-0.590) | 0.549 (0.521-0.577) |
|  | Model 5 | 0.817 | 0.822 | 0.802 | 0.677 (0.651-0.702) | 0.703 (0.677-0.728) | 0.716 (0.692-0.741) |
|  | Model 6 | 0.805 | 0.748 | 0.740 | 0.696 (0.671-0.721) | 0.769 (0.745-0.792) | 0.777 (0.753-0.800) |
|  | Model 7 | 0.807 | 0.773 | 0.756 | 0.690 (0.665-0.715) | 0.754 (0.730-0.778) | 0.760 (0.736-0.784) |
|  | Model 8 | 0.812 | 0.762 | 0.759 | 0.689 (0.664-0.714) | 0.763 (0.739-0.786) | 0.763 (0.739-0.786) |
|  | Model 9 | 0.821 | 0.776 | 0.768 | 0.673 (0.647-0.698) | 0.743 (0.719-0.768) | 0.744 (0.720-0.769) |
|  | Model 10 | 0.832 | 0.770 | 0.737 | 0.667 (0.642-0.693) | 0.752 (0.728-0.776) | 0.771 (0.748-0.794) |

Model 1: Absolute measures at 6, 12, and 24 months. Model 2: Difference between measures at 1-6 months and 6-24 months. Model 3: Difference between measures at 1-12 months and 12-24 months. Model 4: Difference between measures at 1-24 months. Model 5: age and measure at the BMI peak. Model 6: Absolute measures at 6, 12, and 24 months + age and measures at the BMI peak. Model 7: Difference between measures at 1-6 months and 6-24 months + age and measures at the BMI peak. Model 8: Difference between measures at 1-12 months and 12-24 months + age and measures at the BMI peak. Model 9: Difference between measures at 1-24 months + age and measures at the BMI peak. Model 10: Difference between measures at 1 month to BMI peak and BMI peak to 24 months + age and measures at the BMI peak.

CI, confidence intervals; AUC, area under the receiver operating characteristic curve; BMI, body mass index, WfL, weight-for-length.

### Table S3: Residual standard deviation of GECKO-calibrated models at predicting ΔBMI z-score between 2 and 5-7 years of age for each measure of infant growth

| **Calibrated model** | **Weight, kg** | **WfL, kg/cm** | **BMI, kg/m^2^** | **Weight z-score, kg** | **WfL z-score, kg/cm** | **BMI z-score, kg/m^2^** |
| --- | --- | --- | --- | --- | --- | --- |
| Birth model | 0.784 | | | | | |
| Model 1 | 0.776 | 0.769 | 0.639 | 0.774 | 0.648 | 0.637 |
| Model 2 | 0.777 | 0.779 | 0.714 | 0.784 | 0.714 | 0.695 |
| Model 3 | 0.778 | 0.779 | 0.715 | 0.783 | 0.703 | 0.694 |
| Model 4 | 0.782 | 0.779 | 0.776 | 0.785 | 0.778 | 0.777 |
| Model 5 | 0.744 | 0.749 | 0.695 | 0.739 | 0.719 | 0.710 |
| Model 6 | 0.733 | 0.726 | 0.638 | 0.724 | 0.649 | 0.636 |
| Model 7 | 0.736 | 0.736 | 0.658 | 0.728 | 0.663 | 0.906 |
| Model 8 | 0.735 | 0.734 | 0.906 | 0.784 | 0.655 | 0.783 |
| Model 9 | 0.739 | 0.741 | 0.663 | 0.737 | 0.674 | 0.668 |
| Model 10 | 0.757 | 0.756 | 0.646 | 0.738 | 0.668 | 0.642 |
| 2-year Parsimonious Model | 0.635 | | | | | |
| 1-year Parsimonious Model | 0.656 | | | | | |

Model 1: Absolute measures at 6, 12, and 24 months. Model 2: Difference between measures at 1-6 months and 6-24 months. Model 3: Difference between measures at 1-12 months and 12-24 months. Model 4: Difference between measures at 1-24 months. Model 5: age and measure at the BMI peak. Model 6: Absolute measures at 6, 12, and 24 months + age and measures at the BMI peak. Model 7: Difference between measures at 1-6 months and 6-24 months + age and measures at the BMI peak. Model 8: Difference between measures at 1-12 months and 12-24 months + age and measures at the BMI peak. Model 9: Difference between measures at 1-24 months + age and measures at the BMI peak. Model 10: Difference between measures at 1 month to BMI peak and BMI peak to 24 months + age and measures at the BMI peak.

CI, confidence intervals; AUC, area under the receiver operating characteristic curve; BMI, body mass index, WfL, weight-for-length.

### Table S4: Akaike Information Criterion of models at predicting ΔBMI z-score between 2 and 5-7 years of age for each measure of infant growth

| **Calibrated model** | **Weight, kg** | **WfL, kg/cm** | **BMI, kg/m^2^** | **Weight z-score, kg** | **WfL z-score, kg/cm** | **BMI z-score, kg/m^2^** |
| --- | --- | --- | --- | --- | --- | --- |
| Birth model | 11043 | | | | | |
| Model 1 | 8034 | 7953 | 6901 | 8028 | 6938 | 6892 |
| Model 2 | 8067 | 8067 | 7525 | 8146 | 7545 | 7397 |
| Model 3 | 11043 | 11043 | 11043 | 11043 | 11043 | 11043 |
| Model 4 | 8158 | 8096 | 8078 | 8258 | 8097 | 8095 |
| Model 5 | 7674 | 7695 | 7252 | 7651 | 7358 | 7343 |
| Model 6 | 7595 | 7530 | 6890 | 7564 | 6935 | 6891 |
| Model 7 | 7614 | 7558 | 6952 | 7575 | 7083 | 6949 |
| Model 8 | 7613 | 7581 | 6965 | 7576 | 7014 | 6973 |
| Model 9 | 7636 | 7611 | 7068 | 7614 | 7160 | 7120 |
| Model 10 | 7794 | 7788 | 6963 | 7607 | 7078 | 6955 |
| 2-year Parsimonious Model | 6813 | | | | | |
| 1-year Parsimonious Model | 6846 | | | | | |

Model 1: Absolute measures at 6, 12, and 24 months. Model 2: Difference between measures at 1-6 months and 6-24 months. Model 3: Difference between measures at 1-12 months and 12-24 months. Model 4: Difference between measures at 1-24 months. Model 5: age and measure at the BMI peak. Model 6: Absolute measures at 6, 12, and 24 months + age and measures at the BMI peak. Model 7: Difference between measures at 1-6 months and 6-24 months + age and measures at the BMI peak. Model 8: Difference between measures at 1-12 months and 12-24 months + age and measures at the BMI peak. Model 9: Difference between measures at 1-24 months + age and measures at the BMI peak. Model 10: Difference between measures at 1 month to BMI peak and BMI peak to 24 months + age and measures at the BMI peak.

BMI, body mass index, WfL, weight-for-length.

### Table S5: Adjusted R^2^ of models at predicting ΔBMI z-score between 2 and 5-7 years of age for each measure of infant growth

| **Calibrated model** | **Weight, kg** | **WfL, kg/cm** | **BMI, kg/m^2^** | **Weight z-score, kg** | **WfL z-score, kg/cm** | **BMI z-score, kg/m^2^** |
| --- | --- | --- | --- | --- | --- | --- |
| Birth model | 0.089 | | | | | |
| Model 1 | 0.160 | 0.182 | 0.415 | 0.162 | 0.408 | 0.417 |
| Model 2 | 0.151 | 0.151 | 0.286 | 0.129 | 0.281 | 0.314 |
| Model 3 | 0.149 | 0.151 | 0.281 | 0.130 | 0.302 | 0.315 |
| Model 4 | 0.126 | 0.143 | 0.148 | 0.097 | 0.143 | 0.143 |
| Model 5 | 0.252 | 0.247 | 0.347 | 0.258 | 0.324 | 0.327 |
| Model 6 | 0.272 | 0.287 | 0.418 | 0.279 | 0.410 | 0.418 |
| Model 7 | 0.267 | 0.280 | 0.407 | 0.276 | 0.381 | 0.407 |
| Model 8 | 0.267 | 0.274 | 0.404 | 0.276 | 0.394 | 0.403 |
| Model 9 | 0.261 | 0.267 | 0.384 | 0.267 | 0.366 | 0.373 |
| Model 10 | 0.223 | 0.225 | 0.404 | 0.269 | 0.382 | 0.405 |
| 2-year Parsimonious Model | 0.431 | | | | | |
| 1-year Parsimonious Model | 0.335 | | | | | |

Model 1: Absolute measures at 6, 12, and 24 months. Model 2: Difference between measures at 1-6 months and 6-24 months. Model 3: Difference between measures at 1-12 months and 12-24 months. Model 4: Difference between measures at 1-24 months. Model 5: age and measure at the BMI peak. Model 6: Absolute measures at 6, 12, and 24 months + age and measures at the BMI peak. Model 7: Difference between measures at 1-6 months and 6-24 months + age and measures at the BMI peak. Model 8: Difference between measures at 1-12 months and 12-24 months + age and measures at the BMI peak. Model 9: Difference between measures at 1-24 months + age and measures at the BMI peak. Model 10: Difference between measures at 1 month to BMI peak and BMI peak to 24 months + age and measures at the BMI peak.

BMI, body mass index, WfL, weight-for-length.

### Table S6: Performance of models at predicting BMI z-score and overweight at 5-7 years of age for each infant growth measure

| **Cohort** | **Model** | **BMI z-score residual standard deviation** | | | **AUC (95% CI) for predicting overweight** | | |
| --- | --- | --- | --- | --- | --- | --- | --- |
|  |  | **Weight, kg** | **WfL, kg/cm** | **BMI, kg/m^2^** | **Weight, kg** | **WfL, kg/cm** | **BMI, kg/m^2^** |
| ABCD (derivation cohort) | Birth model | 0.907 | | | 0.719 (0.692-0.745) | | |
|  | Model 1 | 0.792 | 0.755 | 0.721 | 0.795 (0.772-0.818) | 0.817 (0.796-0.838) | 0.828 (0.807-0.849) |
|  | Model 2 | 0.835 | 0.848 | 0.875 | 0.776 (0.753-0.800) | 0.771 (0.747-0.795) | 0.754 (0.729-0.779) |
|  | Model 3 | 0.836 | 0.849 | 0.878 | 0.777 (0.754-0.801) | 0.771 (0.747-0.795) | 0.752 (0.726-0.777) |
|  | Model 4 | 0.842 | 0.859 | 0.878 | 0.772 (0.749-0.796) | 0.766 (0.742-0.790) | 0.749 (0.723-0.774) |
|  | Model 5 | 0.854 | 0.833 | 0.731 | 0.757 (0.732-0.783) | 0.763 (0.737-0.789) | 0.823 (0.803-0.844) |
|  | Model 6 | 0.768 | 0.735 | 0.719 | 0.811 (0.789-0.833) | 0.827 (0.806-0.848) | 0.828 (0.807-0.848) |
|  | Model 7 | 0.771 | 0.744 | 0.719 | 0.812 (0.790-0.834) | 0.824 (0.803-0.844) | 0.829 (0.808-0.849) |
|  | Model 8 | 0.771 | 0.743 | 0.721 | 0.812 (0.790-0.834) | 0.823 (0.802-0.843) | 0.828 (0.808-0.849) |
|  | Model 9 | 0.798 | 0.779 | 0.728 | 0.794 (0.772-0.817) | 0.799 (0.777-0.821) | 0.826 (0.805-0.847) |
|  | Model 10 | 0.795 | 0.776 | 0.724 | 0.796 (0.774-0.819) | 0.801 (0.779-0.824) | 0.827 (0.807-0.848) |
| GECKO (external validation cohort) | Birth model | 0.813 | | | 0.638 (0.609-0.666) | | |
|  | Model 1 | 0.769 | 0.748 | 0.743 | 0.691 (0.665-0.717) | 0.721 (0.696-0.747) | 0.762 (0.738-0.785) |
|  | Model 2 | 0.748 | 0.777 | 0.795 | 0.713 (0.688-0.738) | 0.705 (0.680-0.730) | 0.691 (0.664-0.717) |
|  | Model 3 | 0.750 | 0.775 | 0.797 | 0.713 (0.688-0.738) | 0.704 (0.678-0.729) | 0.683 (0.657-0.710) |
|  | Model 4 | 0.753 | 0.781 | 0.797 | 0.708 (0.683-0.734) | 0.701 (0.676-0.727) | 0.675 (0.648-0.702) |
|  | Model 5 | 0.840 | 0.837 | 0.743 | 0.664 (0.637-0.692) | 0.676 (0.649-0.703) | 0.758 (0.734-0.781) |
|  | Model 6 | 0.784 | 0.757 | 0.740 | 0.700 (0.674-0.725) | 0.728 (0.703-0.753) | 0.765 (0.741-0.788) |
|  | Model 7 | 0.780 | 0.758 | 0.736 | 0.704 (0.678-0.730) | 0.731 (0.706-0.756) | 0.764 (0.740-0.787) |
|  | Model 8 | 0.780 | 0.757 | 0.736 | 0.702 (0.676-0.728) | 0.728 (0.703-0.753) | 0.763 (0.739-0.787) |
|  | Model 9 | 0.776 | 0.778 | 0.734 | 0.704 (0.678-0.729) | 0.721 (0.695-0.746) | 0.761 (0.737-0.784) |
|  | Model 10 | 0.777 | 0.776 | 0.737 | 0.706 (0.680-0.731) | 0.722 (0.697-0.747) | 0.762 (0.739-0.786) |

Model 1: Absolute measures at 6, 12, and 24 months. Model 2: Difference between measures at 1-6 months and 6-24 months. Model 3: Difference between measures at 1-12 months and 12-24 months. Model 4: Difference between measures at 1-24 months. Model 5: age and measure at the BMI peak. Model 6: Absolute measures at 6, 12, and 24 months + age and measures at the BMI peak. Model 7: Difference between measures at 1-6 months and 6-24 months + age and measures at the BMI peak. Model 8: Difference between measures at 1-12 months and 12-24 months + age and measures at the BMI peak. Model 9: Difference between measures at 1-24 months + age and measures at the BMI peak. Model 10: Difference between measures at 1 month to BMI peak and BMI peak to 24 months + age and measures at the BMI peak.

CI, confidence intervals; AUC, area under the receiver operating characteristic curve; BMI, body mass index, WfL, weight-for-length.

### Table S7: Performance of models at predicting BMI z-score and overweight at 5-7 years of age for each infant z-score growth measure

| **Cohort** | **Model** | **BMI z-score residual standard deviation** | | | **AUC (95% CI) for predicting overweight** | | |
| --- | --- | --- | --- | --- | --- | --- | --- |
|  |  | **Weight z-score, kg** | **WfL z-score, kg/cm** | **BMI z-score, kg/m^2^** | **Weight z-score, kg** | **WfL z-score, kg/cm** | **BMI z-score, kg/m^2^** |
| ABCD (derivation cohort) | Birth model | 0.907 | | | 0.719 (0.692-0.745) | | |
|  | Model 1 | 0.792 | 0.721 | 0.722 | 0.795 (0.772-0.818) | 0.830 (0.810-0.850) | 0.828 (0.807-0.848) |
|  | Model 2 | 0.884 | 0.872 | 0.869 | 0.743 (0.718-0.768) | 0.753 (0.728-0.778) | 0.757 (0.733-0.782) |
|  | Model 3 | 0.883 | 0.872 | 0.870 | 0.744 (0.719-0.769) | 0.752 (0.727-0.777) | 0.757 (0.732-0.782) |
|  | Model 4 | 0.887 | 0.879 | 0.876 | 0.740 (0.715-0.766) | 0.744 (0.719-0.770) | 0.750 (0.725-0.775) |
|  | Model 5 | 0.853 | 0.815 | 0.752 | 0.761 (0.736-0.787) | 0.783 (0.758-0.807) | 0.814 (0.793-0.835) |
|  | Model 6 | 0.768 | 0.720 | 0.722 | 0.810 (0.788-0.832) | 0.830 (0.810-0.850) | 0.828 (0.807-0.848) |
|  | Model 7 | 0.781 | 0.733 | 0.749 | 0.804 (0.782-0.826) | 0.825 (0.804-0.845) | 0.818 (0.797-0.839) |
|  | Model 8 | 0.780 | 0.733 | 0.749 | 0.807 (0.785-0.829) | 0.826 (0.805-0.846) | 0.819 (0.798-0.840) |
|  | Model 9 | 0.806 | 0.739 | 0.747 | 0.792 (0.769-0.816) | 0.824 (0.803-0.845) | 0.823 (0.803-0.844) |
|  | Model 10 | 0.798 | 0.729 | 0.730 | 0.799 (0.776-0.822) | 0.827 (0.806-0.848) | 0.827 (0.807-0.848) |
| GECKO (external validation cohort) | Birth model | 0.813 | | | 0.638 (0.609-0.666) | | |
|  | Model 1 | 0.771 | 0.732 | 0.740 | 0.689 (0.663-0.715) | 0.759 (0.736-0.783) | 0.762 (0.738-0.785) |
|  | Model 2 | 0.777 | 0.803 | 0.797 | 0.684 (0.657-0.710) | 0.683 (0.656-0.710) | 0.693 (0.666-0.719) |
|  | Model 3 | 0.777 | 0.806 | 0.796 | 0.684 (0.657-0.710) | 0.684 (0.657-0.710) | 0.691 (0.665-0.718) |
|  | Model 4 | 0.777 | 0.809 | 0.797 | 0.677 (0.650-0.703) | 0.667 (0.641-0.694) | 0.679 (0.653-0.706) |
|  | Model 5 | 0.846 | 0.791 | 0.753 | 0.666 (0.639-0.694) | 0.715 (0.689-0.742) | 0.744 (0.720-0.769) |
|  | Model 6 | 0.785 | 0.732 | 0.739 | 0.698 (0.672-0.724) | 0.761 (0.737-0.784) | 0.764 (0.741-0.787) |
|  | Model 7 | 0.790 | 0.746 | 0.752 | 0.696 (0.670-0.721) | 0.751 (0.727-0.775) | 0.746 (0.722-0.770) |
|  | Model 8 | 0.788 | 0.741 | 0.748 | 0.693 (0.667-0.719) | 0.750 (0.726-0.774) | 0.750 (0.726-0.774) |
|  | Model 9 | 0.787 | 0.742 | 0.735 | 0.700 (0.674-0.725) | 0.751 (0.727-0.776) | 0.753 (0.729-0.777) |
|  | Model 10 | 0.783 | 0.736 | 0.736 | 0.703 (0.678-0.729) | 0.757 (0.734-0.781) | 0.764 (0.741-0.787) |

Model 1: Absolute measures at 6, 12, and 24 months. Model 2: Difference between measures at 1-6 months and 6-24 months. Model 3: Difference between measures at 1-12 months and 12-24 months. Model 4: Difference between measures at 1-24 months. Model 5: age and measure at the BMI peak. Model 6: Absolute measures at 6, 12, and 24 months + age and measures at the BMI peak. Model 7: Difference between measures at 1-6 months and 6-24 months + age and measures at the BMI peak. Model 8: Difference between measures at 1-12 months and 12-24 months + age and measures at the BMI peak. Model 9: Difference between measures at 1-24 months + age and measures at the BMI peak. Model 10: Difference between measures at 1 month to BMI peak and BMI peak to 24 months + age and measures at the BMI peak.

CI, confidence intervals; AUC, area under the receiver operating characteristic curve; BMI, body mass index, WfL, weight-for-length.

### Table S8: Residual standard deviation of GECKO-calibrated models at predicting BMI z-score at 5-7 years of age for each measure of infant growth

| **Calibrated model** | **Weight, kg** | **WfL, kg/cm** | **BMI, kg/m^2^** | **Weight z-score, kg** | **WfL z-score, kg/cm** | **BMI z-score, kg/m^2^** |
| --- | --- | --- | --- | --- | --- | --- |
| Birth model | 0.743 | | | | | |
| Model 1 | 0.693 | 0.665 | 0.634 | 0.695 | 0.636 | 0.635 |
| Model 2 | 0.693 | 0.709 | 0.72 | 0.723 | 0.721 | 0.717 |
| Model 3 | 0.694 | 0.708 | 0.724 | 0.723 | 0.722 | 0.718 |
| Model 4 | 0.701 | 0.718 | 0.728 | 0.727 | 0.731 | 0.726 |
| Model 5 | 0.724 | 0.715 | 0.646 | 0.725 | 0.692 | 0.662 |
| Model 6 | 0.685 | 0.658 | 0.634 | 0.689 | 0.635 | 0.634 |
| Model 7 | 0.684 | 0.665 | 0.634 | 0.695 | 0.649 | 0.661 |
| Model 8 | 0.684 | 0.665 | 0.636 | 0.695 | 0.649 | 0.657 |
| Model 9 | 0.690 | 0.681 | 0.641 | 0.697 | 0.650 | 0.653 |
| Model 10 | 0.689 | 0.679 | 0.638 | 0.694 | 0.642 | 0.639 |
| 2-year Parsimonious Model | 0.633 | | | | | |
| 1-year Parsimonious Model | 0.642 | | | | | |

Model 1: Absolute measures at 6, 12, and 24 months. Model 2: Difference between measures at 1-6 months and 6-24 months. Model 3: Difference between measures at 1-12 months and 12-24 months. Model 4: Difference between measures at 1-24 months. Model 5: age and measure at the BMI peak. Model 6: Absolute measures at 6, 12, and 24 months + age and measures at the BMI peak. Model 7: Difference between measures at 1-6 months and 6-24 months + age and measures at the BMI peak. Model 8: Difference between measures at 1-12 months and 12-24 months + age and measures at the BMI peak. Model 9: Difference between measures at 1-24 months + age and measures at the BMI peak. Model 10: Difference between measures at 1 month to BMI peak and BMI peak to 24 months + age and measures at the BMI peak.

CI, confidence intervals; AUC, area under the receiver operating characteristic curve; BMI, body mass index, WfL, weight-for-length.

### Table S9: Coefficients of two Parsimonious risk models for predicting BMI z-score at 5-7 years of age.

|  | 2-year Parsimonious Model | | 1-year Parsimonious Model | |
| --- | --- | --- | --- | --- |
| **Variable** | **Coefficient** | **99% CI** | **Coefficient** | **99% CI** |
| Intercept | 100.509 | 71.470 to 129.547 | 3.615 | 3.300 to 3.930 |
| (Birthweight/10000)^-2^, g | -0.066 | -0.093 to -0.040 | -0.099 | -0.126 to -0.071 |
| (Birthweight/10000)^-2^ × $ln$(Birthweight/10000)), g | -0.027 | -0.039 to -0.016 | -0.040 | -0.052 to -0.028 |
| Male sex, yes | 0.236 | 0.184 to 0.288 | 0.204 | 0.152 to 0.257 |
| Parity, count | -0.056 | -0.085 to -0.026 | -0.064 | -0.093 to -0.034 |
| Middle maternal educational level, yes | -0.111 | -0.197 to -0.024 | -0.145 | -0.234 to -0.056 |
| High maternal educational level, yes | -0.166 | -0.258 to -0.074 | -0.190 | -0.284 to -0.096 |
| Western ethnicity, yes | -0.115 | -0.186 to -0.043 | -0.114 | -0.187 to -0.041 |
| (Pre-pregnancy maternal BMI/10)^2^, kg/m^2^ | 0.201 | 0.151 to 0.250 | 0.205 | 0.155 to 0.255 |
| (Pre-pregnancy maternal BMI /10)^3^, kg/m^2^ | -0.033 | -0.044 to -0.023 | -0.034 | -0.045 to -0.024 |
| Smoking during pregnancy, yes | 0.164 | 0.057 to 0.270 | 0.193 | 0.085 to 0.302 |
| (Weight at 24 months/10000)^-2^, grams | 1.186 | 0.177 to 2.196 | - | - |
| (Weight at 24 months /10000)^3^, grams | 0.349 | 0.238 to 0.460 | - | - |
| (WfL at 24 months/0.1)^-0.5^, kg/cm | -110.836 | -141.234 to -80.438 | - | - |
| ln(WfL at 24 months/0.1), kg/cm | -45.258 | -57.173 to -33.344 | - | - |
| BMI at 12 months/10, kg/m^2^ | 2.137 | 1.758 to 2.515 | - | - |
| BMI at 24 months/10, kg/m^2^ | 1.801 | 1.463 to 2.139 | - | - |
| (BMI at 12 months/10)^-2^, kg/m^2^ | - | - | -10.169 | -10.705 to -9.633 |
| Age at BMI peak between 2 and 365 days, yes | - | - | -0.461 | -0.606 to -0.315 |
| Age at BMI peak > 365 days, yes | - | - | -0.403 | -0.599 to -0.206 |
| Age at BMI peak, days | - | - | 0.001 | 0.000 to 0.001 |

Standard errors are heteroskedasticity robust. The equation for estimating an infant’s BMI z-score at 5-7 years of age is $\beta_{0}+\beta_{1}x_{1}+\beta_{2}x_{2}+\ldots\beta_{n}x_{n}$, where $\beta_{0}$ is the intercept’s coefficient, $\beta_{1}$ to $\beta_{n}$ are the coefficients for each variable in the model (where $n$ is the total number of variables), and $x_{1}$ to $x_{n}$ are the infant’s values for each variable (for continuous variables, insert the corresponding value in the correct units; for categorical variables, insert 1 if the factor is present and 0 if not).

BMI, body mass index; CI, confidence interval, WfL, weight-for-length.

### Table S10: Performance of the two Parsimonious Models and the Birth Model for predicting overweight at 5-7 years of age at a fixed sensitivity threshold of at 0.275

|  | **2-year Parsimonious Model** | | **1-year Parsimonious Model** | | **Birth model** | |
| --- | --- | --- | --- | --- | --- | --- |
| **Performance measure** | **ABCD** | **GECKO** | **ABCD** | **GECKO** | **ABCD** | **GECKO** |
| Residual standard deviation | 0.712 | 0.744 | 0.726 | 0.747 | 0.907 | 0.813 |
| AUC (95% CI) | 0.836 (0.816-0.856) | 0.761 (0.737-0.785) | 0.823 (0.802-0.844) | 0.758 (0.735-0.782) | 0.719 (0.692-0.745) | 0.638 (0.609-0.666) |
| BMI z-score threshold | 1.00 | 0.79 | 0.91 | 0.74 | 0.38 | 0.37 |
| True positives (%) | 112 (65.1) | 138 (63.6) | 112 (58.6) | 138 (57.1) | 111 (35.1) | 138 (40.9) |
| True negatives (%) | 2672 (90.1) | 1619 (81.6) | 2653 (90.0) | 1597 (81.4) | 2527 (89.5) | 1499 (80.4) |
| False positives (%) | 60 (34.9) | 79 (36.4) | 79 (41.4) | 101 (42.3) | 205 (64.9) | 199 (59.1) |
| False negatives (%) | 295 (9.9) | 365 (18.4) | 295 (10.0) | 365 (18.6) | 296 (10.5) | 365 (19.6) |
| Sensitivity | 0.275 | 0.274 | 0.275 | 0.274 | 0.273 | 0.274 |
| Specificity | 0.978 | 0.953 | 0.971 | 0.941 | 0.925 | 0.883 |
| Positive predictive value | 0.651 | 0.636 | 0.586 | 0.577 | 0.351 | 0.409 |
| Negative predictive value | 0.901 | 0.816 | 0.900 | 0.814 | 0.895 | 0.804 |

AUC, area under the receiver operating characteristic curve; BMI, body mass index; CI, confidence interval, NA, not applicable.
